# p53 controls expression of the DNA deaminase APOBEC3B to limit its potential mutagenic activity in cancer cells

**DOI:** 10.1093/nar/gkx721

**Published:** 2017-08-16

**Authors:** Manikandan Periyasamy, Anup K. Singh, Carolina Gemma, Christian Kranjec, Raed Farzan, Damien A. Leach, Naveenan Navaratnam, Hajnalka L. Pálinkás, Beata G. Vértessy, Tim R. Fenton, John Doorbar, Frances Fuller-Pace, David W. Meek, R. Charles Coombes, Laki Buluwela, Simak Ali

**Affiliations:** 1 Department of Surgery & Cancer, Imperial College London, Hammersmith Hospital Campus, London W12 0NN, UK; 2 Department of Pathology, University of Cambridge, Tennis Court Road, Cambridge CB2 1QP, UK; 3 MRC London Institute of Medical Sciences, Imperial College London, Hammersmith Hospital Campus, Du Cane Road, London W12 0NN, UK; 4 Department of Applied Biotechnology and Food Science, Budapest University of Technology and Economics, Budapest 1111, Hungary; 5 Laboratory of Genome Metabolism and Repair, Institute of Enzymology, Research Centre for Natural Sciences, Hungarian Academy of Sciences, Budapest 1117, Hungary; 6 School of Biosciences, University of Kent, Canterbury, Kent CT2 7NJ, UK; 7 Division of Cancer Research, University of Dundee, Ninewells Hospital and Medical School, Dundee DD1 9SY, UK

## Abstract

Cancer genome sequencing has implicated the cytosine deaminase activity of apolipoprotein B mRNA editing enzyme catalytic polypeptide-like (APOBEC) genes as an important source of mutations in diverse cancers, with APOBEC3B (A3B) expression especially correlated with such cancer mutations. To better understand the processes directing A3B over-expression in cancer, and possible therapeutic avenues for targeting A3B, we have investigated the regulation of A3B gene expression. Here, we show that A3B expression is inversely related to p53 status in different cancer types and demonstrate that this is due to a direct and pivotal role for p53 in repressing A3B expression. This occurs through the induction of p21 (CDKN1A) and the recruitment of the repressive DREAM complex to the A3B gene promoter, such that loss of p53 through mutation, or human papilloma virus-mediated inhibition, prevents recruitment of the complex, thereby causing elevated A3B expression and cytosine deaminase activity in cancer cells. As p53 is frequently mutated in cancer, our findings provide a mechanism by which p53 loss can promote cancer mutagenesis.

## INTRODUCTION

The APOBEC3 family of cytosine deaminases are mediators of intrinsic immunity to retroviruses and endogenous retrotransposons, which act by causing cytosine-to-uracil (C-to-U) deamination in single-stranded DNA that is generated during reverse transcription (1,2), to promote deleterious mutations. The seven members of the APOBEC3 gene family (A3A/B/C/D/F/G/H), are related to the APOBEC1, APOBEC2, APOBEC4 and activation-induced deaminase (AID). APOBEC1 functions in RNA editing and DNA editing by AID is required for class-switch recombination and somatic hypermutation of immunoglobulin genes in B cells to augment antibody diversity (3). Additionally, apolipoprotein B mRNA editing enzyme catalytic polypeptide-like (APOBEC) genes, in particular AID, have been implicated in the epigenetic regulation of gene expression by directing the deamination of 5-hydroxymethylcytosine generated by TET enzyme conversion of 5-methylcytosine (for reviews see refs. (4–6)). Here, deamination by AID facilitates base excision repair, resulting in cytosine demethylation. Moreover, we recently reported a DNA methylation-independent role for A3B-mediated cytidine deamination and repair as a mechanism for chromatin remodelling that facilitates estrogen receptor (ER) target gene expression in breast cancer cells (7).

The mutational capacity of APOBECs has led to the proposal that inappropriate and/or upregulated expression of these genes could promote mutations in genomic DNA, a possibility bolstered by the demonstration that AID can cause chromosomal mutations and rearrangements (8–10) and AID, as well as APOBEC1 promote tumourigenesis in transgenic mouse models (11–13). Ectopic expression studies in yeast and mammalian cells have shown that APOBEC3 enzymes can also promote mutations in genomic DNA (14–17). Importantly, sequencing of cancer genomes reveals that a large proportion of somatic mutations in diverse cancer types, including breast, ovarian, cervical, bladder, head and neck and lung cancer, are attributable to APOBEC activity (17–23). A3B is the only one of the 11 APOBEC genes that is consistently expressed at high levels in these cancer types and A3B expression correlates with the number of C-to-T and overall mutational load in cancer genomes (17,21,24). Genome sequencing of yeast cells expressing A3B identify kataegic mutational patterns similar to those that are observed in breast cancer genomes (25) and the incidence of C-to-T mutations in breast cancer cells is reduced by A3B knockdown (17). Together, these studies provide a compelling case for A3B as a driver of the mutational landscape and tumour evolution in many common cancers.

Gene expression analysis shows that A3B levels are low in normal tissues, but are elevated in many cancer types (7,17). Understanding the mechanisms that regulate A3B expression will provide important insights into the processes driving acquisition of cancer mutations and tumour evolution. Originally cloned on the basis of its induction by phorbol ester treatment of normal keratinocytes, A3B expression is stimulated by NF-κB activation by protein kinase C (26,27). Interestingly, mutational signatures associated with A3B activity are especially strong in cervical and head/neck cancers, in which human papillomaviruses (HPV) are important causative agents (20,21,28). Recently, the E6 and E7 viral oncogenes in high-risk HPVs were shown to promote A3B expression (29–32), highlighting a potential explanation for the A3B-associated mutator phenotype in HPV-positive cervical and head/neck cancer. As inhibition of p53 tumour suppressor activity/levels is a key function of E6 (33), we reasoned that p53 might be a direct regulator of A3B expression. Here, we show that p53 represses A3B expression and cytosine deaminase activity in cancer cells, through a p21-dependent mechanism and that loss of p53 activity through its mutation or HPV-16 E6/E7-mediated downregulation, causes A3B upregulation. Further, by assessing cellular cytosine deaminase activity and abasic site generation in genomic DNA, we show that loss of p53 activity through mutation or HPV-directed downregulation can promote increased mutagenic capacity of normal and cancer cells.

## MATERIALS AND METHODS

### Cell lines

Cell lines were obtained from ATCC (LGC Standards, UK) and cultured in Dulbecco's modified Eagle's medium (DMEM) containing 10% foetal calf serum (FCS). HCT116 p53^−/−^ and HCT116 p21^−/−^ cells were kindly provided by Dr B. Vogelstein (34,35). NIKS cell lines have been described previously (36), and were maintained at sub-confluence on γ-irradiated J2–3T3 feeder cells in complete F medium, as described (37). Nutlin-3 (Bio-Techne Ltd, UK) was added to a final concentration of 10 μM, unless otherwise stated. An equal volume of dimethylsulphoxide (DMSO) was added to the vehicle controls. HPV16 E6 and p53-interaction defective E6 mutant, Addgene ID #44152 and 44153, respectively, were a gift from P. Howley (38). The vector, MSCV-N- GFP (Addgene ID: 37855) was a gift from K. Munger (39). Retroviral constructs pLXSN, pLXSN HPV16 E6, pLXSN HPV16 E7 and pLXSN HPV16 E6/E7 were kindly provided by Dr Denise Galloway (40). For the generation of HPV-16 E6-SAT mutant, wild-type (WT) E6 was mutated using PfuUltra DNA polymerase (Stratagene, London, UK) and the following primer pair: E6SAT_forward

5′-GCAATGTTTCAGGACCCACAGGAGAGCGCCACAAAGTTACCACAGTTATGCACAGAGCTGC-3′;

E6SAT_reverse 5′-GCAGCTCTGTGCATAACTGTGGTAACTTTGTGGCGCTCTCCTGTGGGTCCTGAAACATTGC-3′.

### Breast cancer samples

The patients presented with primary, operable breast cancer to the Dundee Cancer Centre between 1997 and 2012 and provided written, informed consent for research use of their tissues. Use of the clinical material and data were approved by the Tayside Tissue Bank under delegated authority from the Tayside Local Research Ethics Committee. Total RNA was extracted from tumour samples using the Qiagen RNA extraction kit and cDNA was prepared using the High Capacity cDNA Reverse Transcription Kit and oligo-dT primers (Applied Biosystems), according to manufacturer’s instructions. Polymerase chain reaction (PCR) with p53-specific primers (5′-TTCCACGACGGTGACACGCT, 5′-CTTCTGACGCACACCTATTG) was used to amplify full length p53 cDNA, followed by Sanger sequencing to identify p53 mutations. Thirty of the 32 mutant samples encoded TP53 mutations/deletions described in the IARC TP53 database (41). Of the remaining samples, one case had a 3-nt deletion causing loss of Ala159 in exon 5, the second featured a single C insertion that would cause a frameshift after Pro309 in exon 9.

### Immunoblotting

Whole cell lysates were prepared in RIPA buffer supplemented with protease and phosphatase inhibitor cocktails (Roche, UK), and immunoblotted as described (7). The A3B antibody has been described previously (7). Antibodies for p21 (sc-397), p53 (sc-126), survivin (BIRC5; sc17779), cyclin B1 (CCNB1; sc-752), E2F4 (sc-866), LIN9 (sc-398234) and HPV16 E7 (sc-6981) were purchased from Santa Cruz Biotechnology Inc. (Germany). ß-actin (ab6276) and MDM2 (ab16895) antibodies were obtained from (Abcam plc, UK). The Lin54 (A303–799A-M) antibody was from Bethyl Laboratories Inc. (USA). HPV16 E6 antibody was from Arbor Vita corporation (CA, USA).

### Real-time RT-PCR (RT-qPCR) and ChIP-qPCR assay

Total RNA was extracted from cells in culture, as described (7). Taqman Gene Expression Assays (Applied Biosystems, UK) were used for RT-qPCR on an ABI 7900HT machine and are detailed in Supplementary Data. Chromatin preparation and immunoprecipitation (ChIP) was performed exactly as described previously (7). Primer sequences for qPCR and ChIP antibodies are listed in Supplementary Data.

### RNA interference

The reverse transfection method using Lipofectamine RNAiMAX (Invitrogen, Thermofisher Scientific, UK) was used with double-stranded RNA oligonucleotides (siRNA), as described previously (7). Nutlin was added after 48 h and RNA and protein lysates were prepared a further 24 or 48 h later, as appropriate. ON-TARGETplus human siRNA for E2F4 (J-003471–12), Lin9 (L-018918–01), Lin54 (L-019325–01), p53 (L-003329–00) and p21 (J-003471–12) were purchased from Dharmacon, Thermofisher Scientific, UK.

### CRISPR-Cas9 for disruption of the TP53 gene

CRISPR-mediated deletion of sequences in the third coding exon of the p53 (TP53) gene was carried out using an Amaxa Type II nucleofector (Lonza, Cologne, Germany) and the MCF7 transfection protocol recommended by the manufacturer. This involved co-transfection of U6 promoter based expression plasmids for CRISPR138077 (5′-ACACCGGCGGCCCCTGCACC-3′) and CRISPR138076 (5′-GGGCAGCTACGGTTTCCGTC-3′), described previously (42) and a hCas9 expression plasmid (a gift from George Church; Addgene plasmid #41815). Following nucleofection, cells were allowed to grow in DMEM supplemented with 10% FCS, and established colonies were screened by PCR for the region encompassing the two CRISPR target sites in TP53 coding exon 3, using primers with the sequences 5′-GATGAAGCTCCCAGAATGCC-3′ and 5′-CACTGACAGGAAGCCAAAGG-3′, where the PCR product for WT and exon 3-deleted TP53 gene is 311 and 226 bp, respectively.

### Cytidine deaminase assay

Cells were lysed in 25 mM HEPES (pH 7.4), 10% glycerol, 150 mM NaCl, 0.5% Triton X-100, 1 mM ethylenediaminetetraacetic acid, 1 mM MgCl_2_ and 1 mM ZnCl_2_, supplemented with protease inhibitors. Lysates were incubated at 37°C for 15 min following addition of 2 μg RNase A (Qiagen). About 1 pmol ssDNA substrate 5′ DY782-ATTATTATTATTATTATTATTTCATTTATTTATTTATTTA-3′ (Eurofins, UK) and 0.75U uracil-DNA glycosylase (NEB) were added to 10 μg protein lysate at 37°C for 1 h. A total of 10 μl 1N NaOH was added and samples incubated for 15 min at 37°C. Finally, 10 μl 1N HCl was added to neutralize the reaction and samples were separated by electrophoresis through 15% urea-polyacrylamide gel electrophoresis gels in Tris-borate-EDTA (1x) at 150V for 2–3 h.

### Apurinic/apyrimidinic (AP) site assay

Genomic DNA was prepared and apurinic/apyrimidinic (AP) site determination was performed using the Oxiselect DNA damage ELISA kit (AP sites) (STA-324), according to manufacturer's protocol (Cell Biolabs Inc. San Diego, CA, USA), using the aldehyde reactive probe (ARP) DNA standards to quantify the number of genomic AP sites.

### Analysis of METABRIC breast cancers and TCGA datasets

The TCGA pan-cancer level-3 somatic mutation and RNA expression data were downloaded from Synapse (https://www.synapse.org/#!Synapse:syn300013). Somatic mutation data for breast cancer (BRCA), lung adenocarcinoma (LUAD) and endometrial cancer (UCEC) from TCGA (43), were segregated according to p53 mutational status and mutational signatures 2 and 13, which have been ascribed to A3B (18), were determined for each tumour type. The Mann–Whitney–Wilcoxon statistical test was used to check the association between p53 mutation status and A3B gene expression and number of APOBEC mutational signatures 2 and 13. Association between p53 mutational status and A3B expression was similarly determined for the 2000 breast cancer samples in METABRIC (44).

## RESULTS

### APOBEC3B expression is repressed by p53

**Figure 1.**
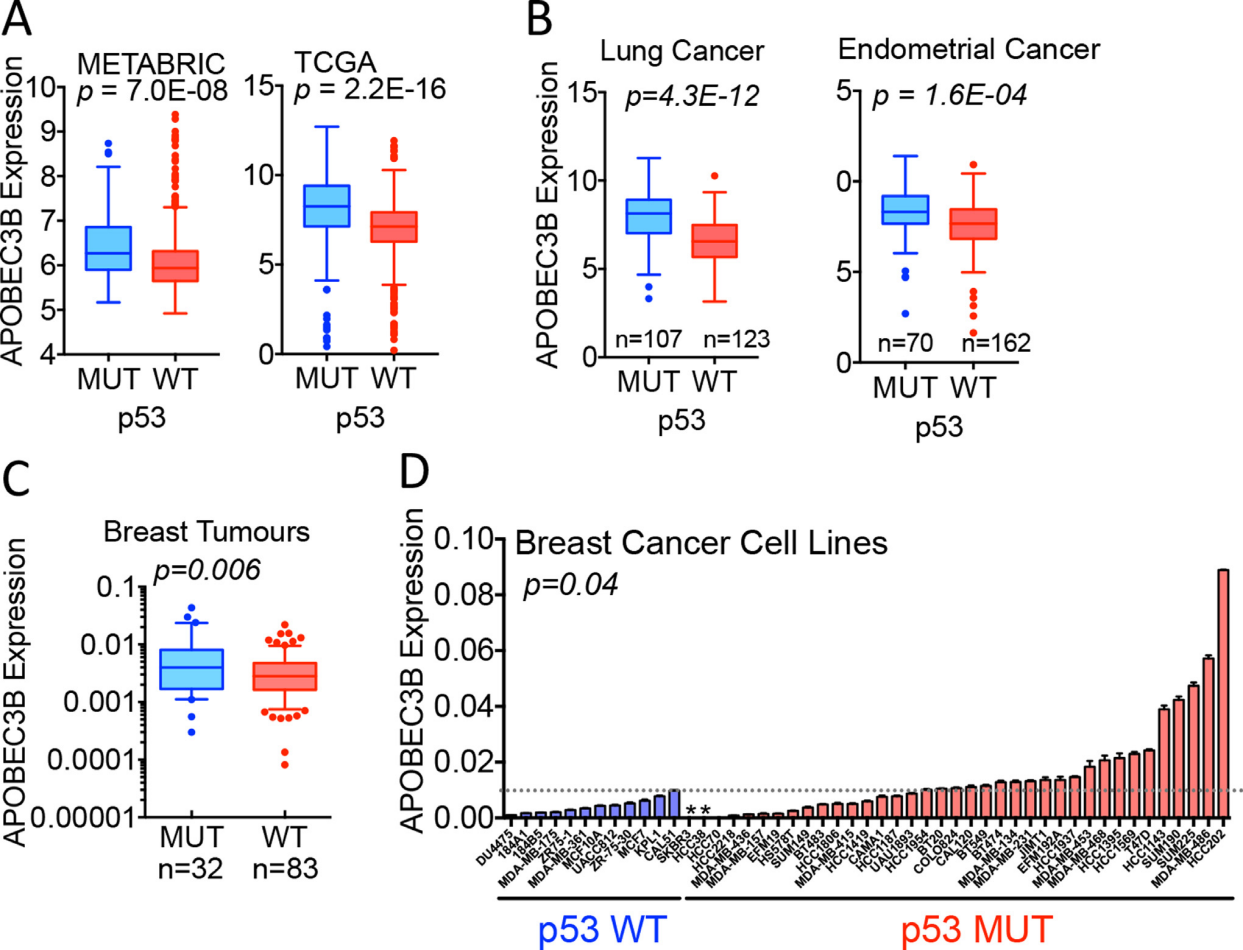
APOBEC3B expression is elevated in breast cancer with mutated p53. (**A**) Analysis of A3B expression in METABRIC and TCGA breast cancer samples. For METABRIC, p53 mutational status was available for 820 samples (*n* = 99 (mutant p53) and 721 (WT p53)). The TCGA dataset comprised 297 samples with mutant and 802 samples with wild-type (WT) p53. (**B**) A3B expression analysis for the TCGA gene expression datasets for lung and endometrial cancer according to p53 mutational status. (**C**) RT-qPCR shows that A3B expression is higher in p53 mutant breast cancers than in p53 WT tumours. A3B expression is shown relative to GAPDH levels. (**D**) RT-qPCR of 50 breast cancer cell lines shows that A3B expression is elevated in cell lines with p53 mutations. A3B expression is shown relative to expression of GAPDH. Asterisks show cell lines (SkBr3, HCC38) that encode the A3A-A3B variant and so do not express A3B.

Previous analyses of gene expression datasets have indicated that A3B levels are elevated in breast cancers with somatic mutations in the p53 gene (TP53), compared with tumours with WT TP53 (17,45,46). In agreement with these findings, we observed a highly significant relationship between A3B expression and p53 mutational status in the METABRIC (*P* = 7.0 × 10^−8^) and TCGA (*P* = 2.2 × 10^−16^) breast cancer cohorts (Figure 1A). An association between A3B expression and p53 mutation was also seen in lung and endometrial cancer (Figure 1B). To further confirm this relationship by RT-qPCR, we analysed gene expression in RNA prepared from 115 primary breast cancers, collected prior to any therapy. A3B expression was significantly greater in p53 mutant tumours (*P* = 0.006; Figure 1C) and was also higher in breast cancer cell lines with mutant, compared with WT p53 (*P* = 0.04; Figure 1D).

**Figure 2.**
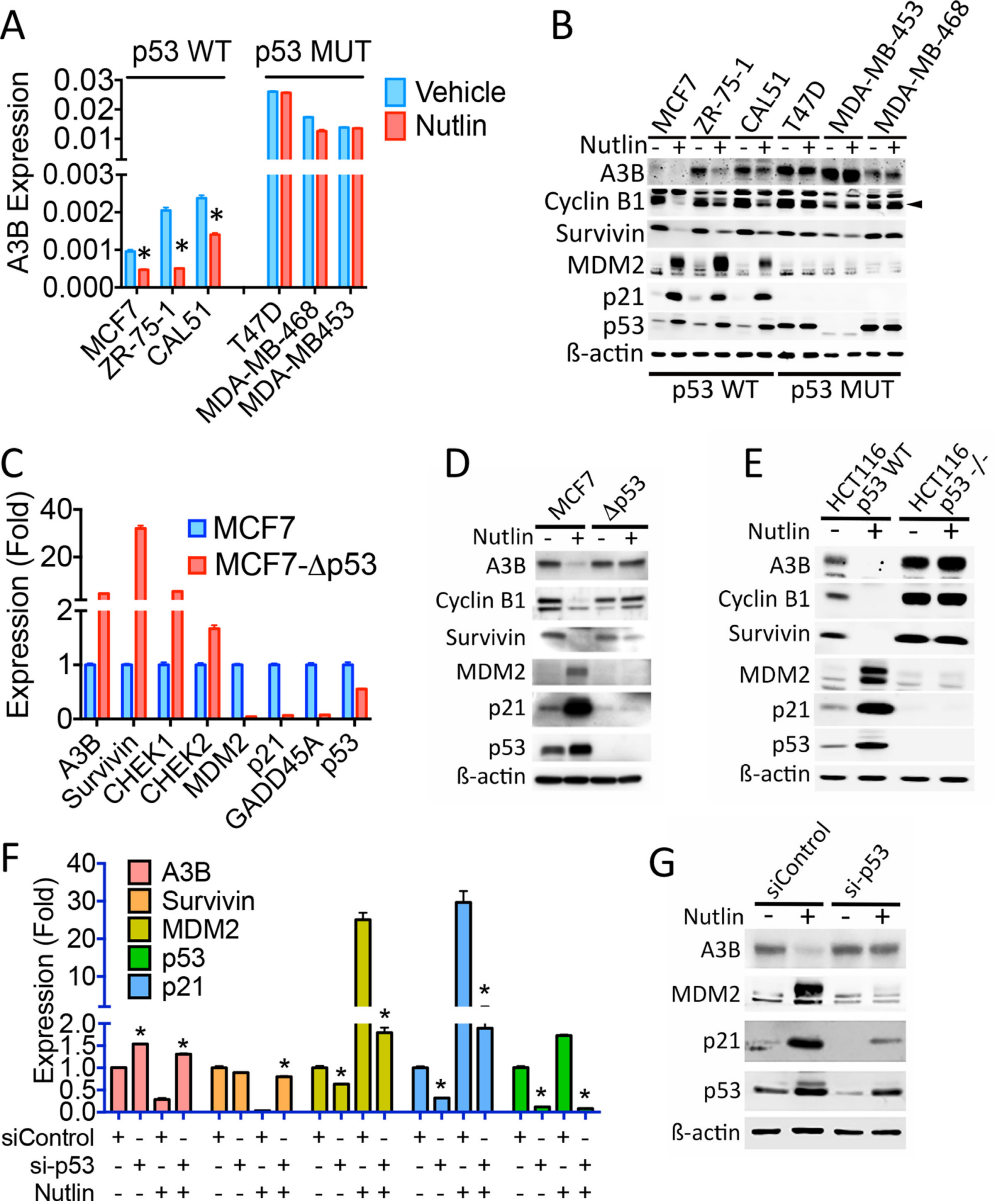
p53 represses APOBEC3B expression in cancer cells. Nutlin (10 μM) was added for 24 h in all experiments. (**A**) RT-qPCR of WT and mutant p53 breast cancer cell lines, treated with Nutlin (*n* = 3). A3B expression is shown relative to GAPDH. Asterisks show significant (*P* < 0.05) differences between vehicle and Nutlin-treated cells. (**B**) Immunoblotting of cell lysates following Nutlin treatment. The filled triangle shows position of Cyclin B1. (**C**) RT-qPCR of MCF7 cells in which exon 3 of the TP53 gene was targeted using CRISPR-Cas9 (MCF7-Δp53). Expression of all examined genes was significantly (*P* < 0.05) different between parental and Δp53 MCF7 cells. (**D**) Immunoblotting of protein lysates from MCF7 and MCF7-Δp53 cells. (**E**) Protein lysates from HCT116 and p53-null HCT116 (HCT116-p53^−/−^) cells ± Nutlin. (**F**) Twenty-four hours following transfection of HCT116 cells with siRNA for p53, Nutlin was added. RT-qPCR was performed using RNA prepared 48 h following addition of Nutlin. (**G**) Immunoblotting of HCT116 cells transfected with si-p53.

In order to further understand the link between p53 status and A3B expression, we looked for evidence of A3B regulation by p53. Treatment with the p53 activator Nutlin-3 (hereafter referred to as Nutlin) was found to reduce A3B expression in breast cancer cell lines with WT p53 (Figure 2A and B). Nutlin did not affect A3B expression in breast cancer cells with p53 mutations. Specificity of Nutlin action on A3B was indicated by the fact that A3B repression occurred over a Nutlin dose range and time course that repressed other well-characterized p53-repressed genes (survivin, CHEK1, CHEK2) (47) and which induced expression of p53-activated genes (MDM2, p21) (Supplementary Figures S1 and 2). Expression of A3B and other p53-repressed genes was elevated and there was almost complete loss in expression of p53-activated genes in MCF7 cells in which the p53 gene was inactivated by CRISPR-Cas9 genome editing (Figure 2C and D). We note that despite appreciable levels of p53 mRNA, there was no detectable p53 protein in MCF7 cells following CRIPSR-Cas9-mediated deletion of exon 3. A3B expression was also elevated in p53-null HCT116 colon cancer cells (HCT116-p53^−/−^), compared with the isogenic line expressing WT p53 (Figure 2E). Moreover, Nutlin did not inhibit A3B expression in p53-null HCT116 cells. Finally, siRNA-mediated p53 knockdown prevented A3B repression by Nutlin (Figure 2F and G). These experiments demonstrate clearly that A3B expression is regulated by p53 and indicate that loss of p53 activity due to its mutation, results in elevated expression of A3B.

### p53 regulation of APOBEC3B expression is mediated by the E2F4/RB-containing DREAM repressive complex

p53 ChIP following Nutlin treatment of HCT116 cells showed enrichment of p53 at the MDM2, GADD45A and CDKN1A (p21) gene promoters, but not at the A3B promoter (Supplementary Figure S3A). Analysis of available ChIP-seq datasets for several cell lines also failed to provide evidence for p53 binding within 20 kb of the A3B gene transcription start site, as exemplified for U2OS cells (48) (Supplementary Figure S3B). There was also absence of p53 at promoters of other p53-repressed genes such as the survivin (BIRC5) gene. By contrast, Nutlin strongly promoted p53 recruitment to the MDM2 and p21 gene promoters.

**Figure 3.**
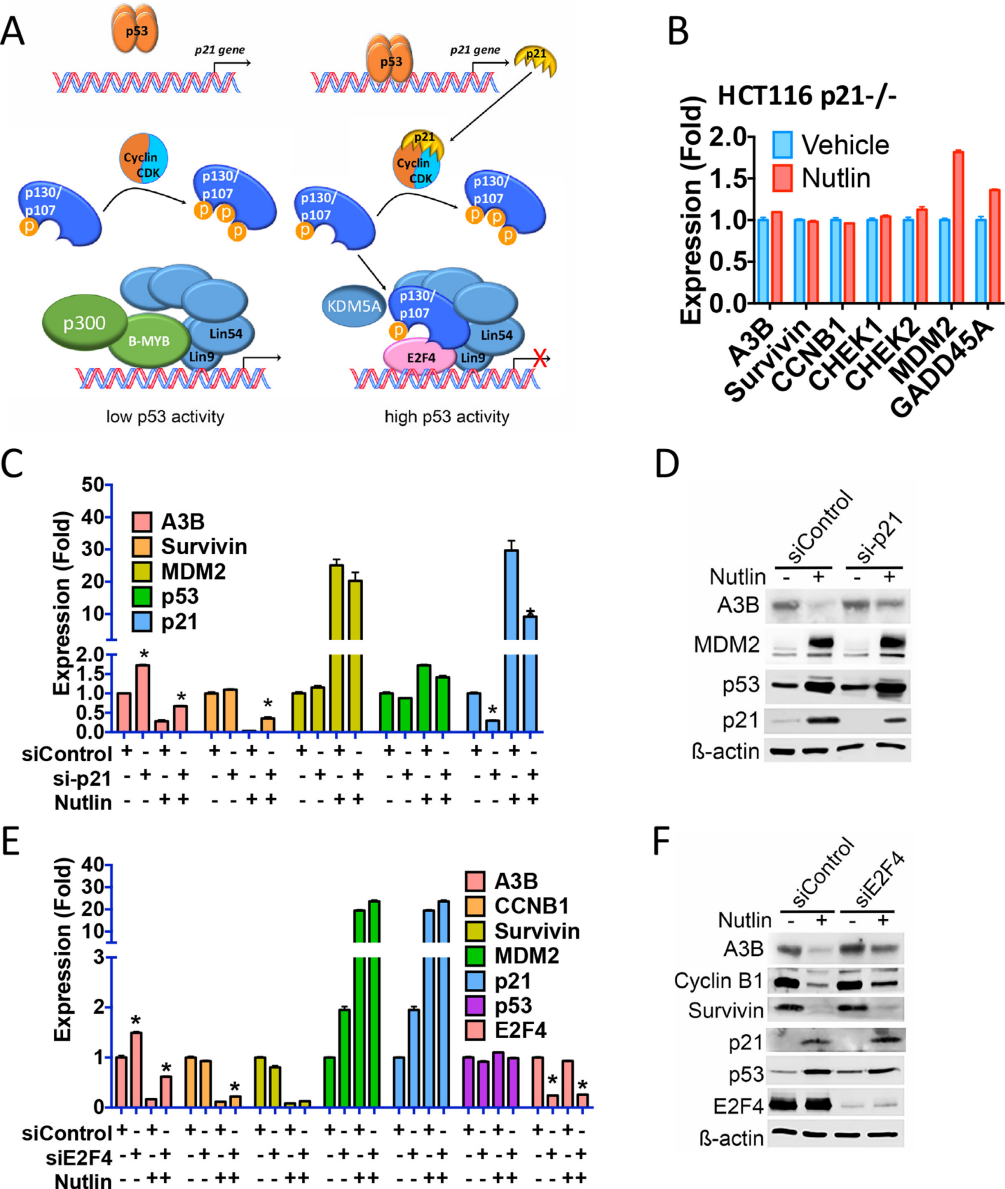
p53 regulation of APOBEC3B expression is mediated by p21 acting through the E2F4 DREAM transcriptional complex. (**A**) Shown is a model depicting the mechanism by which the DREAM complex regulates gene expression following p53 activation. (**B**) RT-qPCR for p21-null HCT116 cells treated with Nutlin for 24 h. (**C**) RT-qPCR of HCT116 cells transfected with p21 siRNA. Nutlin was added for 24 h. Significant (*P* < 0.05; *n* = 3) differences between vehicle and Nutlin-treated cells are highlighted by asterisks. (**D**) Immunoblotting of HCT116 cell lysates following p21 knockdown. (**E** and **F**) RT-qPCR and immunoblotting of HCT116 cells transfected with siRNA for E2F4.

Previous studies have shown that p53 induces expression of target genes by direct recruitment to gene promoters, whereas gene repression by p53 is indirect, frequently involving p21 (47). p21-directed inhibition of cyclin-dependent kinases prevents hyperphosphorylation of p107/p130 retinoblastoma (RB) proteins, promoting the conversion of the so-called multi-B-MYB-multi-vulval class B transcription activation complex, to the E2F4/p107/p130-containing DREAM (dimerization partner, RB-like, E2F and MuvB) repressive complex (Figure 3A) (49). In p21-null HCT116 cells, Nutlin did not repress expression of A3B or other p53-repressed genes (Figure 3B). Inhibition of A3B expression was also blunted by siRNA-mediated p21 knockdown (Figure 3C and D). Inhibition of A3B expression by Nutlin was alleviated by E2F4 knockdown (Figure 3E and F), and was also rescued by siRNA for Lin9 and Lin54, which are key components of the DREAM complex (Supplementary Figure S4).

**Figure 4.**
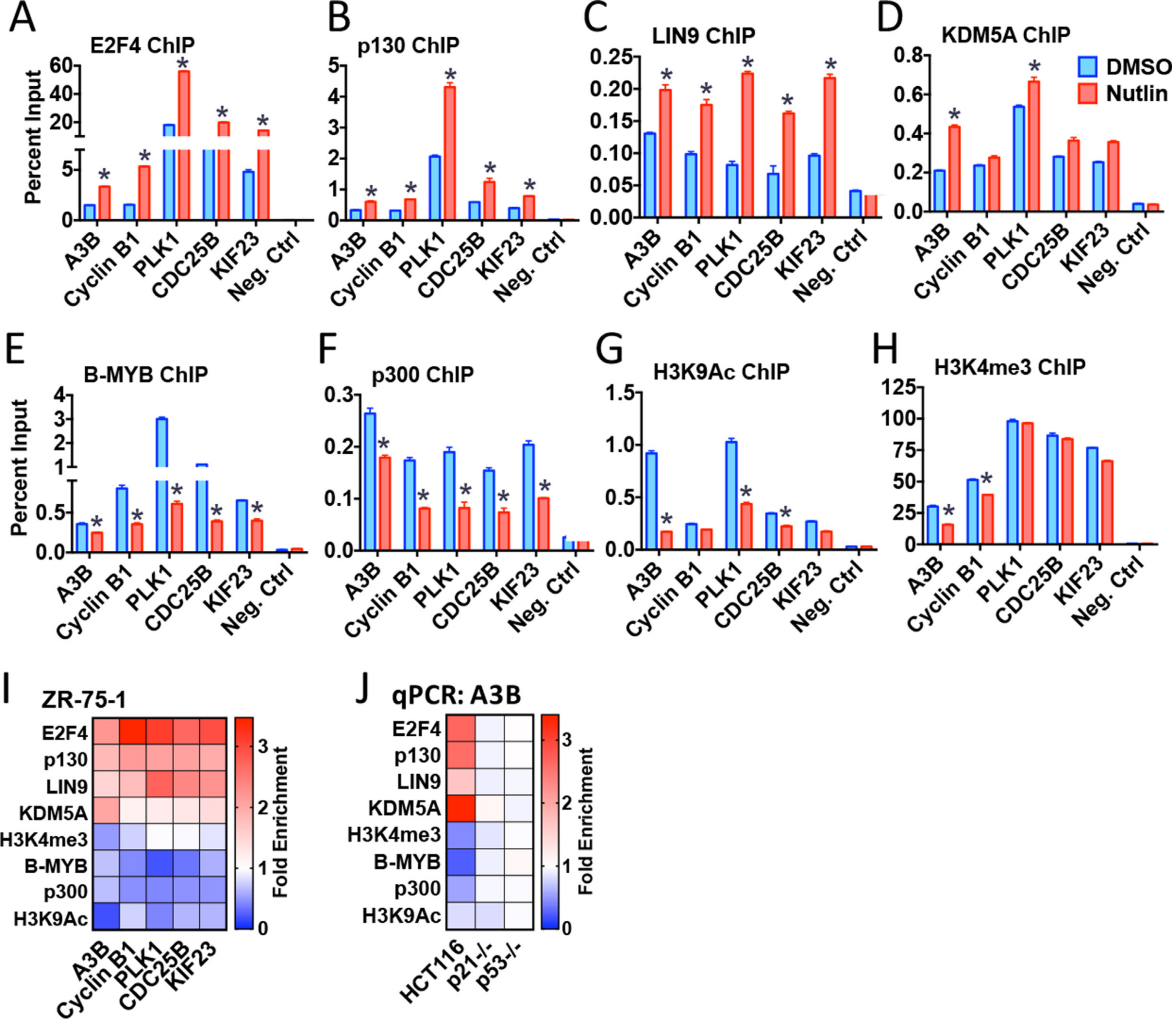
ChIP analysis of DREAM complex enrichment at the APOBEC3B gene promoter. (**A**–**H**) ZR-75–1 cells were treated with Nutlin (10 μM, 24 h), followed by ChIP for transcription factors in the DREAM complex and for histone marks associated with active genes. Asterisks identify significant (*P* < 0.05; *n* = 3) differences in transcription factor recruitment and histone marks for the Nutlin-treated cells, relative to vehicle controls. (**I**) ChIP-qPCR for Nutlin-treated samples are shown, as fold enrichment relative to vehicle. (**J**) ChIP-qPCR for the A3B gene in HCT116 cells, in p21-null or in p53-null HCT116 cells. The heat map shows Nutlin-promoted changes in factor recruitment to the A3B gene, relative to vehicle controls. The full ChIP-qPCR data are shown in Supplementary Figure S5.

ChIP, followed by real-time PCR (ChIP-qPCR), showed that Nutlin stimulates E2F4, p130 and LIN9 recruitment to the A3B gene promoter, with concomittant loss of the transcriptional activators B-MYB and p300 (Figure 4A–F; summarized in the heat map in Figure 4I). The Nutlin-stimulated transition from the activation complex to the repressive complex was accompanied by reduction in the histone marks, H3K9Ac and H3K4me3, both of which are high at the transcription start sites of active genes (Figure 4G and H; Supplementary Figure S3B). KDM5A/JARID1A, originally identified as a protein that binds to RB, contributes to the repression of E2F4 target genes by removing di- and tri-methyl groups from H3K4 (50,51). ChIP for KDM5A showed Nutlin stimulated KDM5A recruitment to the A3B promoter (Figure 4D), explaining the reduction in H3K4me3. Nutlin treatment similarly promoted E2F4, p130, LIN9 and KDM5A recruitment to the A3B promoter and reduced B-MYB, p300, H3K4me3 and K3K9Ac in HCT116 cells (Figure 4J and Supplementary Figure S5A). The dependence on p53 for the Nutlin stimulated gain of E2F4/p130/LIN9/KDM5A at the A3B gene promoter was confirmed in p53-null HCT116 cells (Figure 4J and Supplementary Figure S5B). Furthermore, there was no reduction in B-MYB or p300 enrichment at the A3B gene promoter in HCT116-p53^−/−^ cells; nor was there a reduction in levels of histone H3 marks associated with active transcription. The importance of p21 in directing the recruitment of the DREAM complex was confirmed by the fact that its Nutlin promoted enrichment at the A3B gene promoter was prevented in p21-null HCT116 cells (Figure 4J and Supplementary Figure S5C).

### p53 inhibits the mutational capacity of cancer cells by repressing APOBEC3B expression

**Figure 5.**
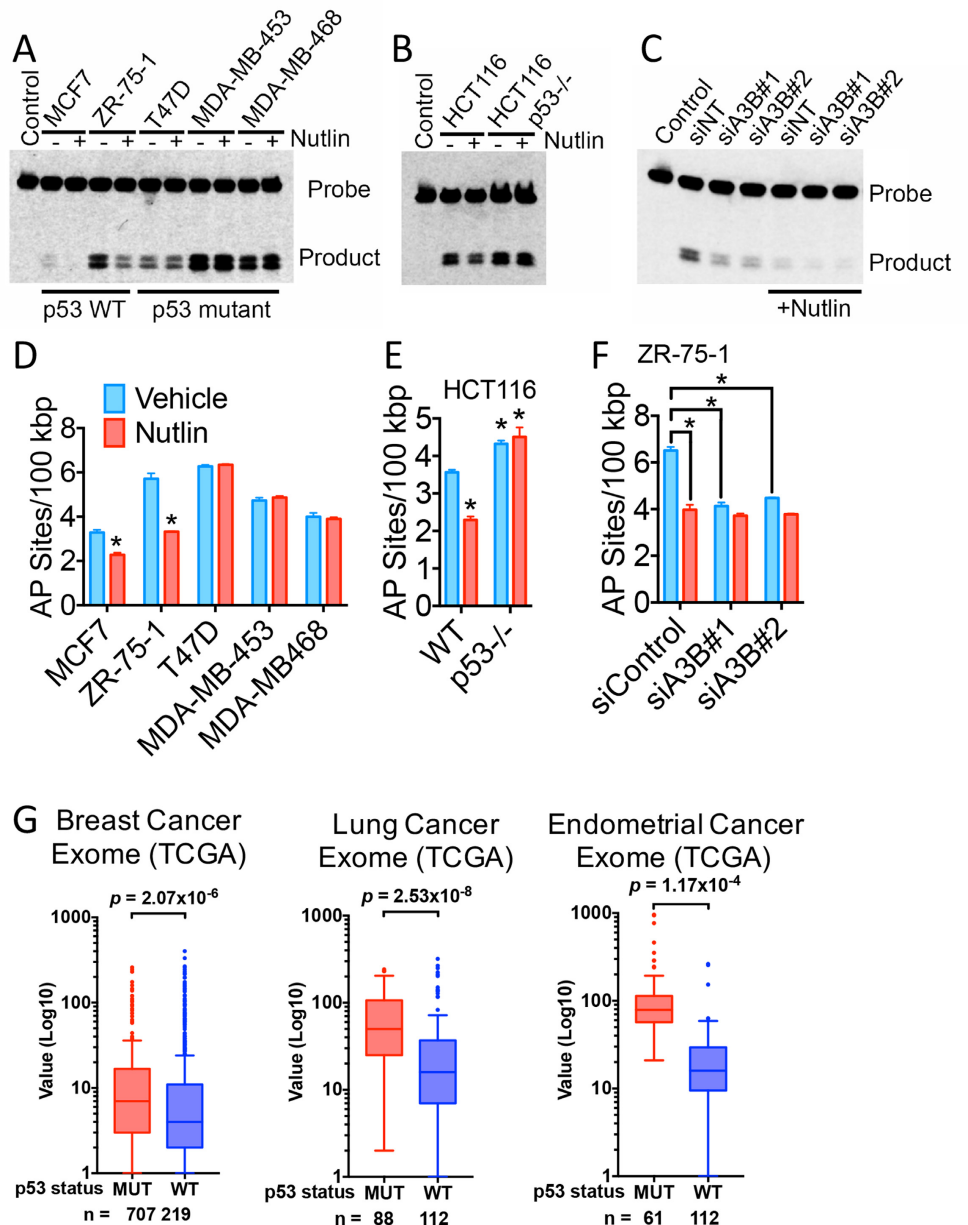
Inhibition of APOBEC3B expression by p53 controls mutagenesis in cancer cells. (**A**and**B**) Protein lysates prepared from Nutlin**-**treated cells were used in an *in vitro* cytidine deaminase assay. Positions of the substrate (probe) and the deamination product, are labelled. (**C**) Cytidine deamination with lysates prepared from ZR-75–1 cells, following transfection with two independent A3B siRNAs. (**D**–**F**) AP or abasic sites in genomic DNA were biotin labelled by conversion with an aldehyde reactive probe (ARP) and quantification of biotinylated DNA. Asterisks show significant (*P* < 0.05; *n* = 3) differences in AP sites for Nutlin-treated samples, relative to vehicle controls. (**G**) Analysis of A3B mutational signature for WT and mutant p53 in breast, lung and endometrial cancer from TCGA. Values on the y-axis represent the number of A3B mutational signatures 2 and 13 in each cancer. *P*-values were calculated using Mann–Whitney–Wilcoxon statistical test.

The above results show that p53 represses A3B expression by directing the E2F4/p107/p130-containing DREAM complex to the A3B promoter and predict that p53 controls A3B expression to limit its mutagenic potential. Indeed, cytosine deaminase activity was generally higher in extracts from mutant p53 lines than in lysates from cells with WT p53 (Figure 5A and Supplementary Figure S6A), consistent with the elevated A3B expression levels in breast cancer cells with mutant p53. Importantly, Nutlin reduced cytidine deaminase activity in lysates from WT-p53 MCF7 and ZR-75–1 cells, but not in those from mutant p53 cells. The p53-dependence of Nutlin-mediated reduction in cytosine deaminase activity was confirmed in HCT116 and p53-null HCT116 cells (Figure 5B and Supplementary Figure S6B). Note that cytosine deaminase activity was strongly reduced by A3B siRNA (Figure 5C and Supplementary Figure S6C), consistent with the fact that A3B is the main APOBEC expressed in breast cancer cells (7,17).

Cytosine deamination by APOBECs generates U:G mismatches that are excised by uracil DNA glycosylases, to generate apurinic/abasic sites that can be processed by AP endonuclease (52). The aldehyde group on the open ring of AP sites can be labelled with an ‘ARP’ containing biotin, thus allowing detection and quantification of AP sites in the genome (53). Interestingly, Nutlin treatment led to reductions in genomic AP sites in cells with WT-p53, but not in cells with p53 mutations (Figure 5D). Nor did Nutlin affect AP sites in p53-null HCT116 cells (Figure 5E). Furthermore, in ZR-75–1 cells, A3B knockdown reduced AP sites and Nutlin did not further inhibit AP sites, indicating that the reduction in abasic sites is almost entirely mediated by the Nutlin/p53 regulation of A3B (Figure 5F).

Sequencing of tumour DNAs representing diverse cancer types, has identified patterns of DNA base alterations that are characteristic of the enzymatic activity of A3B (17–23). Interrogation of exome sequence datasets from the TCGA database showed that the frequency of mutations associated with the A3B mutational signatures was significantly higher for mutant p53 tumours compared with WT-p53 in breast (*P* = 2.07 × 10^−6^), lung (*P* = 2.53 × 10^−8^) and endometrial cancer (*P* = 0.048) (Figure 5G). In conclusion, p53 restricts the mutagenic activity of A3B by repressing its expression, and loss of p53 in cancer results in elevated A3B expression, which may promote somatic mutagenesis in cancer.

### HPV E6 and E7 gene-mediated repression of p53 and the E2F4/p107/p130-containing DREAM complex promotes APOBEC3B expression and activity

**Figure 6.**
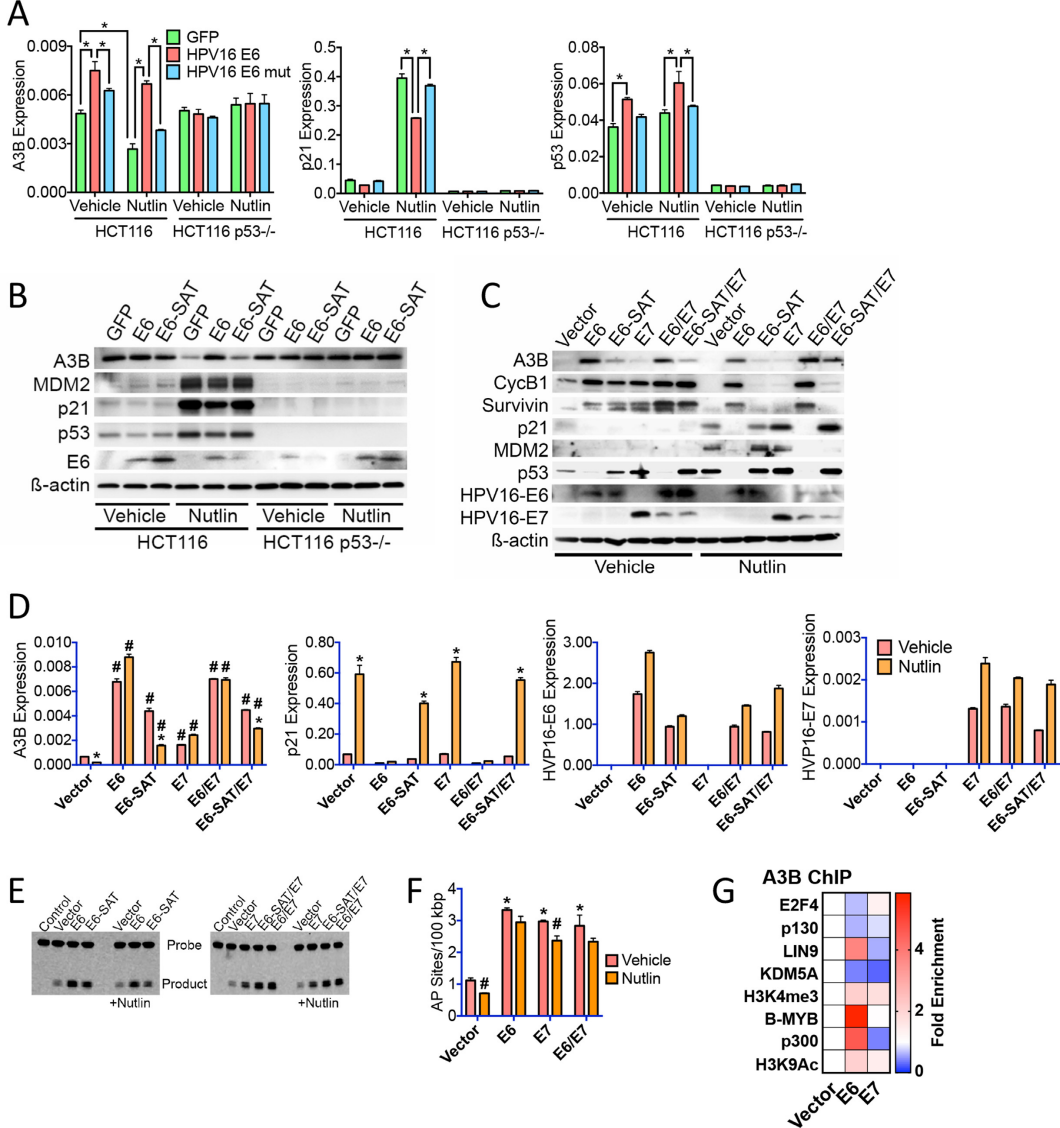
HPV16 E6 and E7 viral oncogenes promote APOBEC3B expression and activity by inhibiting the action of the E2F4/p107/p130 DREAM transcriptional repression complex. (**A**) HCT116 and p53-null HCT116 cells were transfected with HPV16 E6 or the E6-SAT mutant that does not interact with p53. RT-qPCR was performed using RNA prepared 24 h following addition of Nutlin (10 μM). Asterisks show significant (*P* < 0.05; *n* = 3) differences in mRNA expression. (**B**) Cells treated as above, were processed for immunoblotting. (**C**) Normal immortalized human keratinocyte line (NIKS) stably transduced with E6, the E6-SAT mutant, E7, E6/E7, E7/mutant E6 or vector only, were treated with Nutlin (10 μM) for 24 h, followed by preparation protein lysates for immunoblotting. (**D**) NIKS were treated as in C, followed by preparation of total RNA and RT-qPCR. Asterisks show significant (*P* < 0.05) reduction in mRNA expression for Nutlin-treated samples relative to the vehicle treated NIKS for each transduced line. Cross-hatches show significant (*P* < 0.05) difference in mRNA expression relative to the vehicle treated and vector transduced NIKS. Results for three experiments are shown. (**E**) The *in vitro* cytidine deaminase assay was used to assess A3B activity in protein lysates prepared 24 h following addition of 10 μM Nutlin to the E6 and E7 transduced NIKS cells. (**F**) Quantification of biotin-labelled ARP conversion of abasic sites in genomic DNA from E6 and E7 transduced NIKS cells. Asterisks show significant (*P* < 0.05) differences in AP sites compared to vehicle-treated vector transduced NIKS; cross-hatches denote statistically significant reduction in AP sites by Nutlin treatment for each transduction. (**G**) ChIP-qPCR for vector, E6 or E7 transduced NIKS cells is shown as fold change in transcription factor/histone mark enrichment at the A3B gene promoter, relative to vehicle controls. The actual factor enrichment relative to input is shown in Supplementary Figure S7B.

Given that p53 downregulation and Rb family inactivation are key functional targets of the high-risk HPV E6 and E7 genes (33), we ascertained whether the transcriptional mechanism for p53-mediated repression of A3B expression identified here is subverted by these viral oncogenes. Indeed, A3B expression was elevated in HPV16 E6 transfected HCT116 cells, but was unaffected by E6 in p53-null HCT116 cells (Figure 6A and B). Stimulation of A3B expression was prevented in cells transfected with an E6 mutant that is defective for interaction with p53 (54). Moreover, E6, but not mutant E6, abrogated repression of A3B by Nutlin. Consistent with these effects being mediated by inhibition of p53 action by E6, p21 expression was reduced by WT, but not mutant E6.

To further evaluate the mechanisms of E6 and E7 regulation of p53 activity and consequence for A3B expression, we repeated the analysis in human keratinocytes, the natural HPV target cell. For this purpose we used normal immortalized human keratinocytes (NIKS), a spontaneously immortalized but not transformed keratinocyte cell line (55), stably expressing E6, E7, E6 and E7 (E6/E7) (36). In agreement with the HCT116 results, p53 expression was abrogated in NIKS transduced with E6, but not mutant E6 (Figure 6C). This was accompanied by an increase in A3B and a reduction in expression of the p53-induced p21 and MDM2 genes (Figure 6C and D). E6 expression also prevented the Nutlin-mediated repression of A3B, survivin and cyclin B1, as well as induction of p21 and MDM2. In concordance with the described role for E7 in inhibiting the activity of Rb family members, expression of A3B and the other p53-repressed genes was elevated in E7-expressing NIKS, but E7 did not affect direct p53 targets (p21, MDM2). In this context, the inhibitory effect of HPV-16 E7 on p21 might contribute to the enhanced expression of p53-repressed genes (56,57). A recent meta-analysis of global HPV E7-regulated gene expression profiling datasets indicates that HPV-16 E7 can indeed activate expression of DREAM complex-repressed genes (58), including that of A3B (31,58), confirming our findings. However, E7 expression had a notably milder effect on A3B upregulation, compared to E6 (Figure 6C and D). This is consistent with previous studies in NIKS, where the HPV-16 E7-mediated inhibition of RB family members and effects on cell growth are alleviated by presence of growth factors in culture medium (36). Cytosine deaminase activity was elevated in NIKS expressing E6 and/or E7 (Figure 6E), as was the number of abasic sites detectable in genomic DNA (Figure 6F), consistent with elevated A3B expression and activity.

As observed in other cell types, p53 was not recruited to the A3B gene in NIKS (Supplementary Figure S7A). In agreement with its role in inhibiting p53, there was reduced p53 recruitment at the MDM2 and p21 promoters in E6 expressing NIKS, but not in cells expressing E7. B-MYB and p300 recruitment, as well as histone methylation and acetylation associated with gene activity, were elevated by E6 (Figure 6G). Conversely, recruitment of the repressive DREAM complex (E2F4, p130), as well as KDM5A, was reduced. Whilst B-MYB and p300 recruitments were not increased by E7, recruitment of the repressors p130 and KDM5A was reduced at the A3B promoter, accompanied by increased H3K4 tri-methylation. Similar results were obtained for the other p53-repressed genes analysed (Supplementary Figure S7). Taken together, these results demonstrate that stimulation of A3B expression by HPV E6 and E7 oncogenes is mediated by inhibition of the p53 directed transcriptional repression and the stimulation of transcriptional activation by the B-MYB/E2F4/p107/p130 DREAM complex.

## DISCUSSION

APOBEC3 genes play important roles in innate immunity and act by causing hypermutation of retroviral genomes and are implicated in reducing retrotransposon mobilization. Their mutational activities bring an inherent risk to the host genome, as shown by ectopic expression of APOBEC3 genes, which can demonstrably cause genomic mutations (14–17). Therefore, to safeguard genomic integrity, tight regulation of APOBEC expression is necessary in order to suppress their mutagenic potential. Indeed, there is a strong evidence that over-expression of A3B causes hypermutation in many cancer types. We observed that A3B expression is higher in breast cancers with mutant p53 than in those with WT p53, in agreement with previous reports (17,45,46). We have found that a similar relationship between A3B expression and p53 status can be extended to lung and endometrial cancer, cancer types in which the mutational landscape is marked by mutations consistent with A3B activity (20,21,23). These findings originally suggested that A3B might be involved in the mutation of the p53 gene. Indeed, A3B over-expression in breast cancer cell lines has been reported to promote cytosine deamination in the p53 gene (17). However, the majority of p53 mutations in cancer do not appear to conform to the TCW DNA motif targeted by A3B, we investigated whether A3B expression is regulated by p53 (ref. (59)), although as has been pointed out, p53 mutations detected in tumours will not simply reflect the exonic nucleotide sequence, but the selection imparted by the resultant amino acid change (60).

Notwithstanding, we show here that p53 controls APOBEC3B expression. Loss of p53 by siRNA-mediated knockdown, gene disruption or HPV E6 expression, increases A3B expression, while Nutlin activation of p53 represses A3B expression in multiple cell types, strong evidence that p53 is a negative regulator of A3B. Several mechanisms directing repression of p53 target genes have been reported, including direct binding of p53, or indirect recruitment via interaction with other transcription factors such as NF-Y (47,61,62). Despite the presence of potential p53 response element sequences in the A3B gene (48), analysis of diverse p53 ChIP-seq datasets provides little evidence for p53 recruitment to the A3B promoter. Indeed, in only one out of eight genome-wide p53 binding datasets analysed by Menendez *et al.* (48) was there evidence for p53 binding at the A3B promoter, who also reported weak Nutlin- and doxorubicin-induced p53 recruitment to the A3B gene (48). Moreover, we did not observe enrichment of p53 at the A3B promoter in ZR-75–1, HCT116 or NIKS, arguing against an important role for direct or indirect p53 recruitment to the A3B promoter. Furthermore, p53 activation did not repress A3B expression in p21-null HCT116 cells and p21 knockdown rescued A3B repression by Nutlin, demonstrating the importance of p21 in the repression of A3B by p53.

The MuvB complex, together with B-MYB drives expression of many cell-cycle regulated genes through the S phase and G2/M, recruitment to the cell cycle genes homology region (CHR) element being mediated by the LIN54 component of MuvB (49,63), whereas MuvB, together with E2F4 and p107/p130, known as the DREAM complex, represses expression of these genes. E2F4 and p107/p130 recruitment to this complex is promoted by p21-mediated inhibition of CDK directed p107/p130 hyperphosphorylation. ChIP experiments have demonstrated (i) the presence of B-MYB and the MuvB subunit LIN9 at the A3B promoter, together with the B-MYB associating p300 histone acetyltransferase, (ii) a reduction in B-MYB and p300 recruitment upon Nutlin treatment, accompanied by stimulation of E2F4 and p130 recruitment, as well as the RB-associated histone lysine demethylase KDM5A, (iii) together with a reduction in the levels of histone marks associated with active transcription. The role of the B-MYB/MuvB complex is confirmed by a recent study which showed that B-MYB is a transcriptional driver of A3B expression and that B-MYB and A3B expression is correlated in many cancer types (64). We further show that deletion of p53 or of p21 prevented Nutlin-mediated loss of B-MYB/p300 or gain of E2F4/p130/KDM5A. HPV16 E6 abrogated p21 expression, resulting in reduced E2F4/p130/KDM5A recruitment and promoting B-MYB and p300 binding and stimulation of activation histone marks at the A3B promoter. E7 also inhibited recruitment of p130 and KDM5A, consistent with action of E7 in inhibiting gene repression by RB proteins (33).

The presence of E2F4, LIN9, LIN54 and p130 at the A3B promoter are confirmed in global ChIP–chip experiments (49,65), and identify a region in the A3B promoter encoding a potential cell-cycle-dependent element and a CHR element, 5′-GGGAGGtcacTTTAAG-3′ ∼50 bp upstream of the A3B transcription start site. Interestingly, expression of the TEA domain (TEAD) transcription factors and their co-activators YAP/TAZ is stimulated by HPV16 E6 directed degradation of p53 and YAP/TAZ/TEAD promotes A3B expression (32). Taken together, these findings further support a critical role for p53 in regulating A3B expression, with repression of A3B expression by the p53-mediated displacement of the MYB-MuvB transcription activation complex by the E2F4/p107/p130 DREAM transcriptional repression complex. We would speculate that p53 also displaces the YAP/TAZ/TEAD complex from the A3B promoter by repressing YAP, TAZ and TEAD expression.

Nutlin treatment results in high level expression of the direct p53 target gene, the MDM2 E3 ubiquitin ligase. MDM2 interacts with pRb to promote its ubiquitination and proteasomal degradation (66,67). Nutlin treatment also stimulates MDM2-dependent accumulation of hypophosphorylated pRb (68). Although MDM2 also interacts with p107 and p130, it does not promote their ubiuitination or proteasomal degradation (67). E2F1 also interacts with MDM2 and Nutlin regulates proteasomal degradation of E2F1 (69,70). However, we failed to observe an effect of Nutlin on E2F4 protein levels, arguing against a role for Nutlin and/or MDM2 driven proteasomal degradation of p107/p130 or E2F4 in p53-mediated repression of A3B expression.

It is nonetheless likely that other signals impact on A3B expression by acting to alter the expression and/or activities of one or more of the factors in the MuvB and/or DREAM complexes. For example, it has recently been shown that the PI3K/AKT pathway promotes gene expression by phosphorylating KDM5A, to relocalize it to the cytoplasm (71), which would provide a potential mechanism for the described high level A3B expression/activity associated with HER2 amplification and/or PTEN loss (72). Further, co-expression analysis of breast and other cancer types demonstrated was reported to show strong enrichment for mitosis and cell-cycle-associated functional ontology groups, with A3B expression (46), which is in keeping with the established role of MuvB and DREAM complexes in the regulating expression of cell cycle and mitosis genes (49,63).

In response to genotoxic and non-genotoxic insult that challenges genomic integrity, p53 mediates innate tumour suppression by altering expression of genes to favour biological functions such as cell cycle arrest, apoptosis and senescence. Our results indicate that p53 also protects the genome by limiting the mutational potential of genes whose primary cellular role is in mutagenic inactivation of foreign and mobilizable DNA. We have shown here that failure to suppress A3B expression following mutational or viral inactivation of p53, results in elevated A3B expression and activity, with attendant increase in potential genetic mutations, as demonstrated by the ability of activated WT, but not mutant, p53 to suppress abasic site generation in genomic DNA and through the demonstration that the frequency of mutations ascribed to A3B activity in diverse cancer types is elevated in p53 mutant tumours. Interestingly, in its recently identified role in regulating the expression of ER target genes in breast cancer cells (7), elevated A3B expression could promote transcriptional programs that aid breast cancer progression, which is consistent with association between high A3B expression and poor patient survival in ER-positive breast cancer (7,46,73) and response to the anti-estrogen tamoxifen (74). Thus, elevated A3B expression and activity due to p53 inactivation likely have important consequences for tumour development and tumour evolution, including response to therapies, both through its role in transcription and because of its potential for inflicting mutational damage.

## Supplementary Material

Supplementary Data
