# Supplementary material for: p53 controls expression of the DNA deaminase APOBEC3B to limit its potential mutagenic activity in cancer cells
Source: Nucleic Acids Res. 2017 Aug 16;45(19):11056–69. doi: 10.1093/nar/gkx721 (PMC5737468; doi:10.1093/nar/gkx721)
Supplement: Supplementary Data [file gkx721_supp.zip › nar-01454-d-2017-File008.docx]

**p53 controls cellular mutagenesis by repressing expression of the DNA deaminase APOBEC3B**

Manikandan Periyasamy^1^, Anup K. Singh^1^, Carolina Gemma^1^, Christian Kranjec^2^, Raed Farzan^1^, Damian A. Leach^1^, Naveenan Navaratnam^3^, Hajnalka L. Pálinkás^4,5^, Beata G. Vértessy^4,5^, Tim R. Fenton^6^, John Doorbar^2^, Frances Fuller-Pace^7^, David W. Meek^7^, R. Charles Coombes^1^, Laki Buluwela^1^, Simak Ali^1, *^

^1^Department of Surgery & Cancer, Imperial College London, Hammersmith Hospital Campus, London W12 0NN, UK.

^2^Department of Pathology, University of Cambridge, Tennis Court Road, Cambridge CB2 1QP, UK.

^3^MRC Clinical Sciences Centre, Imperial College London, Hammersmith Hospital Campus, Du Cane Road, London W12 0NN, UK.

^4^Department of Applied Biotechnology and Food Science, Budapest University of Technology and Economics, Budapest, 1111, Hungary.

^5^Laboratory of Genome Metabolism and Repair, Institute of Enzymology, Research Centre for Natural Sciences, Hungarian Academy of Sciences, Budapest, 1117, Hungary.

^6^School of Biosciences, University of Kent, Canterbury, Kent CT2 7NJ, UK.

^7^Division of Cancer Research, University of Dundee, Ninewells Hospital and Medical School, Dundee DD1 9SY, UK.

^*^Author for correspondence: Simak Ali, Department of Surgery & Cancer, Imperial College London, Du Cane Road, London W12 0NN, UK

Email: [simak.ali@imperial.ac.uk](mailto:simak.ali@imperial.ac.uk)

**Supplementary Information Figure Legends**

**Supplementary Figure S1. p53 represses APOBEC3B expression in ZR-75-1 breast cancer cells.** (**A, C**) Nutlin-3 was added to ZR-75-1 cells at the concentrations shown. RNA and protein lysates were prepared after 24 hours. (**B, D**) Nutlin-3 (10 µM) was added to ZR-75-1 cells. RNA and protein lysates were prepared at the time points shown. (**A, B**) RT-qPCR is shown relative to GAPDH levels (n=3). RT-qPCR was done using ABI gene expression assays for GAPDH (Hs99999905), A3B (Hs00358981), p21 (CDKN1A, Hs00355782), survivin (BIRC5, Hs04194392), MDM2 (Hs01066930), CHEK1 (Hs00967506) and CHEK2 (Hs0020048). (**C, D**) Immunoblotting of cell lysates following Nutlin treatment.

**Supplementary Figure S2. p53 represses APOBEC3B expression in HCT116 colon cancer cells.** All treatments and sample processing was carried out as in Supplementary Figure S1. RT-qPCR results are shown for three replicates.

**Supplementary Figure S3. Repression of A3B expression by p53 does not involve its recruitment to the A3B gene promoter.** (**A**) p53 ChIP following addition of 10 µM Nutlin for 24 hours to HCT116 cells (n=3). Real-time PCR was carried out with primers having the following sequences: A3B (5’-GGTCACTTTAAGGAGGGCTGT-3’, 5’-TAGATACGCTTGTCCCTGTCC-3’), MDM2 (5’-GTTCAGTGGGCAGGTTGACT-3’, 5’-CACTGAACACAGCTGGGAAA-3’), GADD45A (5’-ACTTTCAGCCGAGATGTGCT-3’, 5’-GAGTAGCTGGGCTGACTGCT-3’) and CDKN1A (p21) (5’-CTCCATCCCTATGCTGCCTG-3’, 5’-AGGCAGCCCAAGGACAAAAT-3’). NEU3 (5’-TCTTTTCGTTGCCGTTACCT-3’, 5’-TGGGGAGACTGAGACAGAGG-3’) was chosen from analysis of p53 ChIP-seq data sets and served as a negative control for p53 binding. Antibody for p53 ChIP was purchased from Santa Cruz Biotechnology Inc, Germany (cat. No.: sc-126). (**B**) Genome browser snapshots show the p53 activated genes MDM2 and CDKN1A (p21), as well as the APOBEC3B and BIRC5 (surviving) genes. p53 ChIP-seq data for U2OS cells treated for 24 hours with 10 µM Nutlin was downloaded from NCBI GEO (GEO accession number: [GSE46642](https://www.ncbi.nlm.nih.gov/geo/query/acc.cgi?acc=GSE46642)). Other ChIP-seq data sets are from the ENCODE project (https://www.encodeproject.org).

**Supplementary Figure S4. Repression of A3B expression by the p53-p21-E2F4/RB DREAM complex.** HCT116 cells were transfected with Dharmacon ON-TARGETplus human Lin9 (L-018918-01) or Lin54 (L-019325-01) siRNAs. Nutlin was added 48 hours following transfection. (**A**) Total RNAs prepared a further 24 hours later were used for RT-qPCR. Gene expression was normalized to GAPDH expression and is shown as fold relative to the vehicle treated siControl transfections. Asterisks denote p<0.05 for three replicates. RT-qPCR was performed using ABI gene expression assays for GAPDH (Hs99999905), A3B (Hs0035898), p21 (CDKN1A, Hs00355782), MDM2 (Hs01066930), cyclin B1 (CCNB1, Hs01030099), p53 (TP53, Hs01034249), Lin9 (Hs00542748) and Lin54 (Hs00418240). (**B**) Immunoblotting of HCT116 protein lysates prepared from Nutlin treated cells following transfection with Lin9 and Lin54 siRNAs, as in **A**.

**Supplementary Figure S5. ChIP analysis for the DREAM complex in wild-type HCT116, p21 null HCT116 and p53 null HCT116 cells shows that p53 and p21 are required from the switch from the activating DREAM complex to the repressive DREAM complex.** ChIP-qPCR of HCT116 (**A**), HCT116 p53-/- (**B**) and HCT116 p21-/- (**C**) cells treated with 10 µM Nutlin for 24 hours. Asterisks show significant (p<0.05, n=3) differences in factor enrichment for Nutlin treated samples, relative to vehicle treated cells. ChIP was performed using antibodies for E2F4 (sc-866), p130 (sc-317), Lin9 (sc-398234), B-MYB (sc-724) and p300 (sc-584) from Santa Cruz Biotechnology. Antibodies for KDM5A (A300-897A), H3K9ac (61251) and H3K4me3 (ab8580) were purchased from Bethyl, Active Motif and Abcam, rexpectively. Real-time PCR of ChIP was performed using primers for amplifying the promoter regions of APOBEC3B (5’-GGTCACTTTAAGGAGGGCTGT-3’, 5’-TAGATACGCTTGTCCCTGTCC-3’), p21 (CDKN1A) (5’-CTCCATCCCTATGCTGCCTG-3’, 5’-AGGCAGCCCAAGGACAAAAT-3’), MDM2 (5’-GTTCAGTGGGCAGGTTGACT-3’, 5’-CACTGAACACAGCTGGGAAA-3’), GADD45A (ACTTTCAGCCGAGATGTGCT, GAGTAGCTGGGCTGACTGCT), cyclin B1 (CCNB1) (5’-ATCTAACCCGCTACCCCATT-3’, 5’-AGCCAGGGAAGCCTCTAGTT-3’), CDC25B (5’-ATCTAACCCGCTACCCCATT-3’, 5’-AGCCAGGGAAGCCTCTAGTT-3’), KIF23 (5’-AACCCTCAGTTTCGTCATCG-3’, 5’-GAAGTGGCGCTTGTCTCCTA-3’) and CNTNAP5 (negative control) (5’-TCTCACCTCACTGCAACCAC-3’, 5’-TAGTTGGGCATGGTGTCTCA-3’). Accompanying Figure 4J.

**Supplementary Figure S6. Inhibition of APOBEC3B expression by p53 controls mutagenesis in cancer cells.** (**A**) Immunoblotting of protein lysates from breast cancer cell lines, accompanying the *in vitro* cytidine deamination assay shown in Fig. 5A. (**B**) Immunoblotting of protein lysates from HCT116 and HCT116 p53-/-, accompanying the *in vitro* cytidine deamination assay in Fig. 5B. (**C**) RT-qPCR of ZR-75-1 cells following transfection with two independent siRNAs for A3B. Accompanying Fig. 5C.

**Supplementary Figure S7. ChIP-qPCR analysis for the influence of HPV16 E6 and E7 viral oncogenes on E2F4/p107/p130 DREAM transcriptional complex recruitment to p53 target genes.** (**A**) p53 ChIP and qPCR using chromatin lysates prepared from NIKS transduced with HPV16 E6, E7 or with empty vector. (**B**) ChIP-qPCR for the named transcription factors or histone marks using chromatin prepared from E6 and E7 transduced NIKS. Accompanying Figure 6G. Asterisks represent statistically significant (p<0.05) differences in factor enrcihment, relative to vector controls (n=3).
