# Supplementary material for: p53 controls expression of the DNA deaminase APOBEC3B to limit its potential mutagenic activity in cancer cells
Source: Nucleic Acids Res. 2017 Aug 16;45(19):11056–69. doi: 10.1093/nar/gkx721 (PMC5737468; doi:10.1093/nar/gkx721)
Supplement: Supplementary Data [file gkx721_supp.zip › nar-01454-d-2017-File009.pptx]

## Slide 1
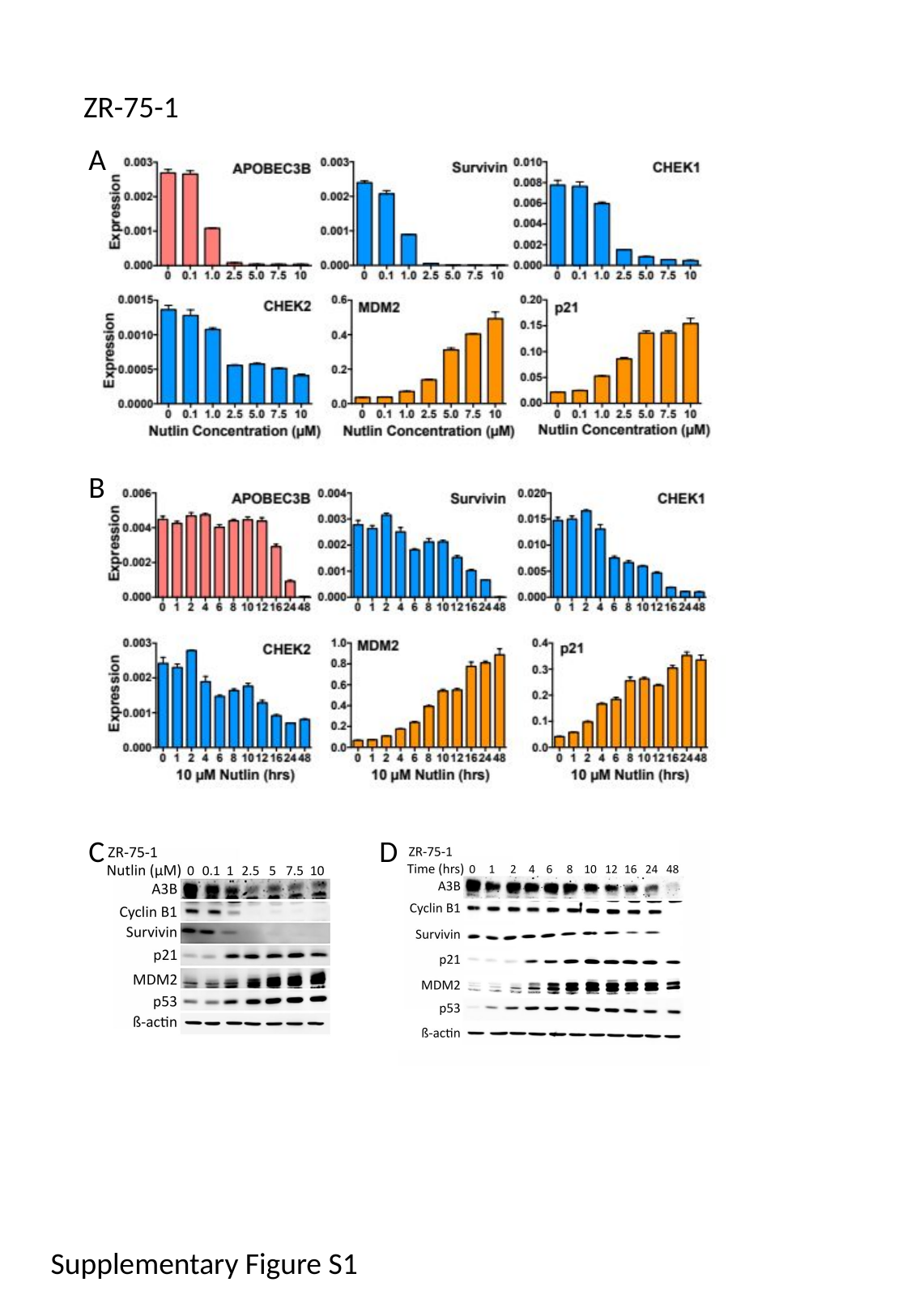

ZR-75-1
A
B
C D
Supplementary Figure S1

## Slide 2
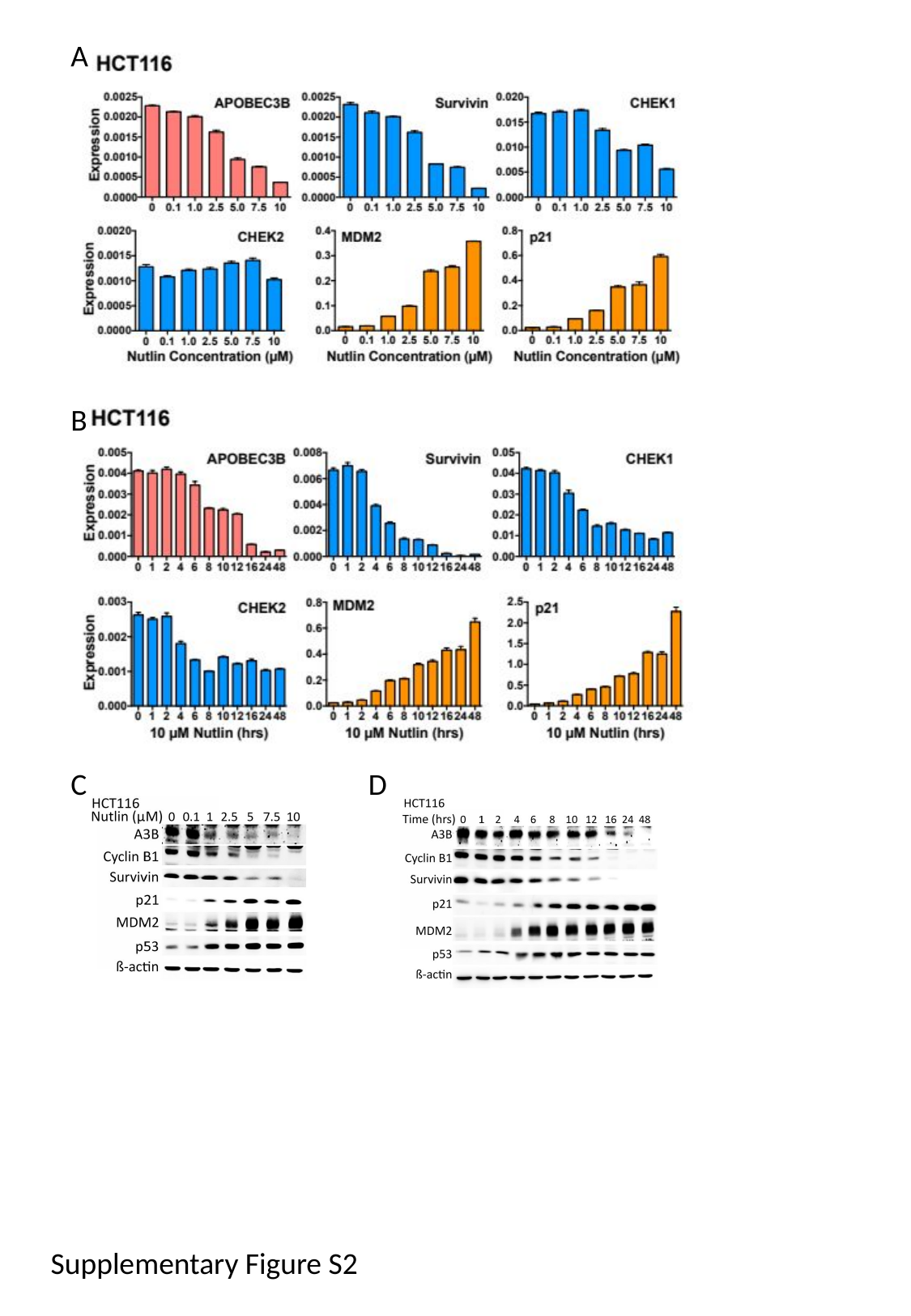

A
B
C D
Supplementary Figure S2

## Slide 3
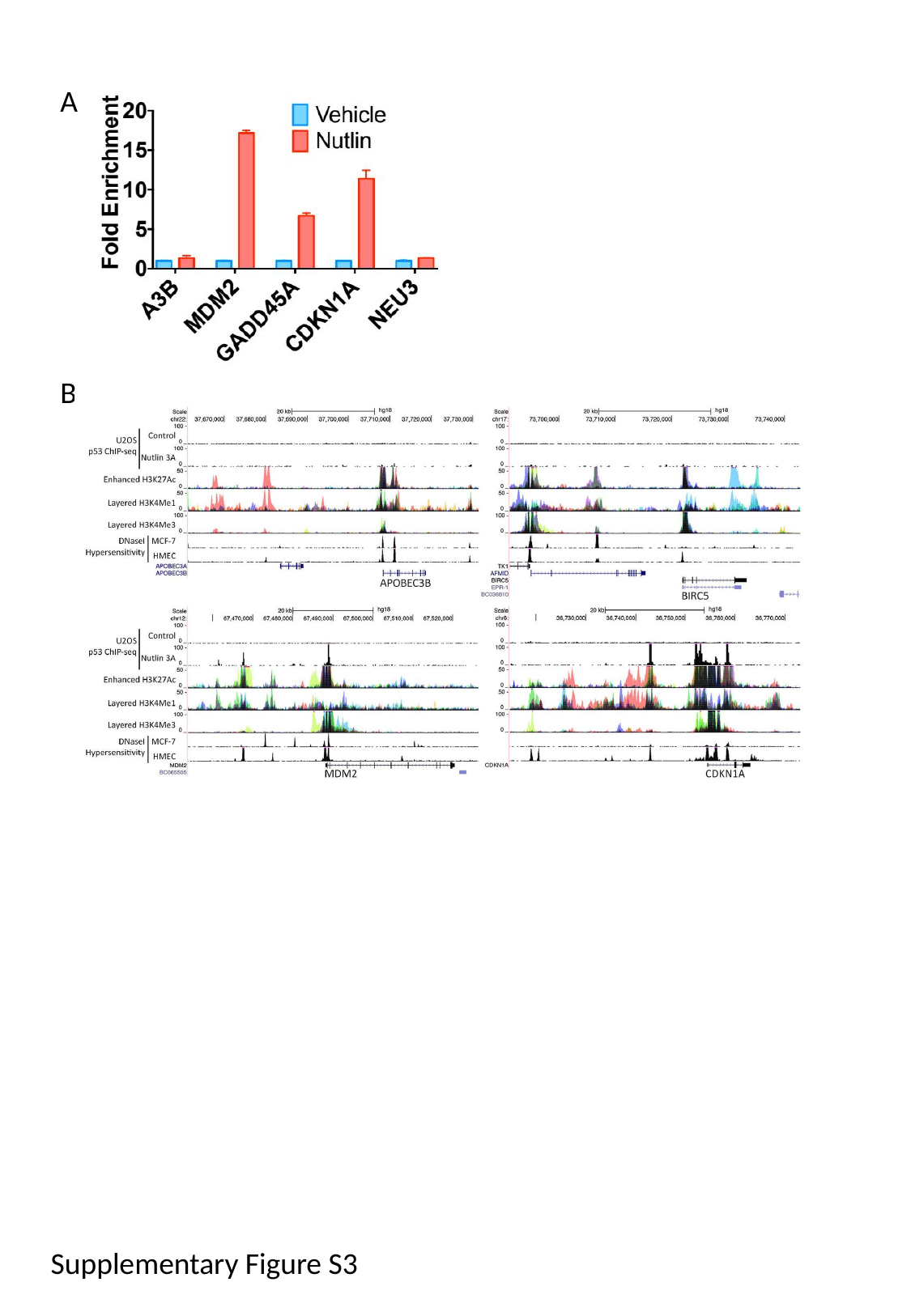

A
B
Supplementary Figure S3

## Slide 4
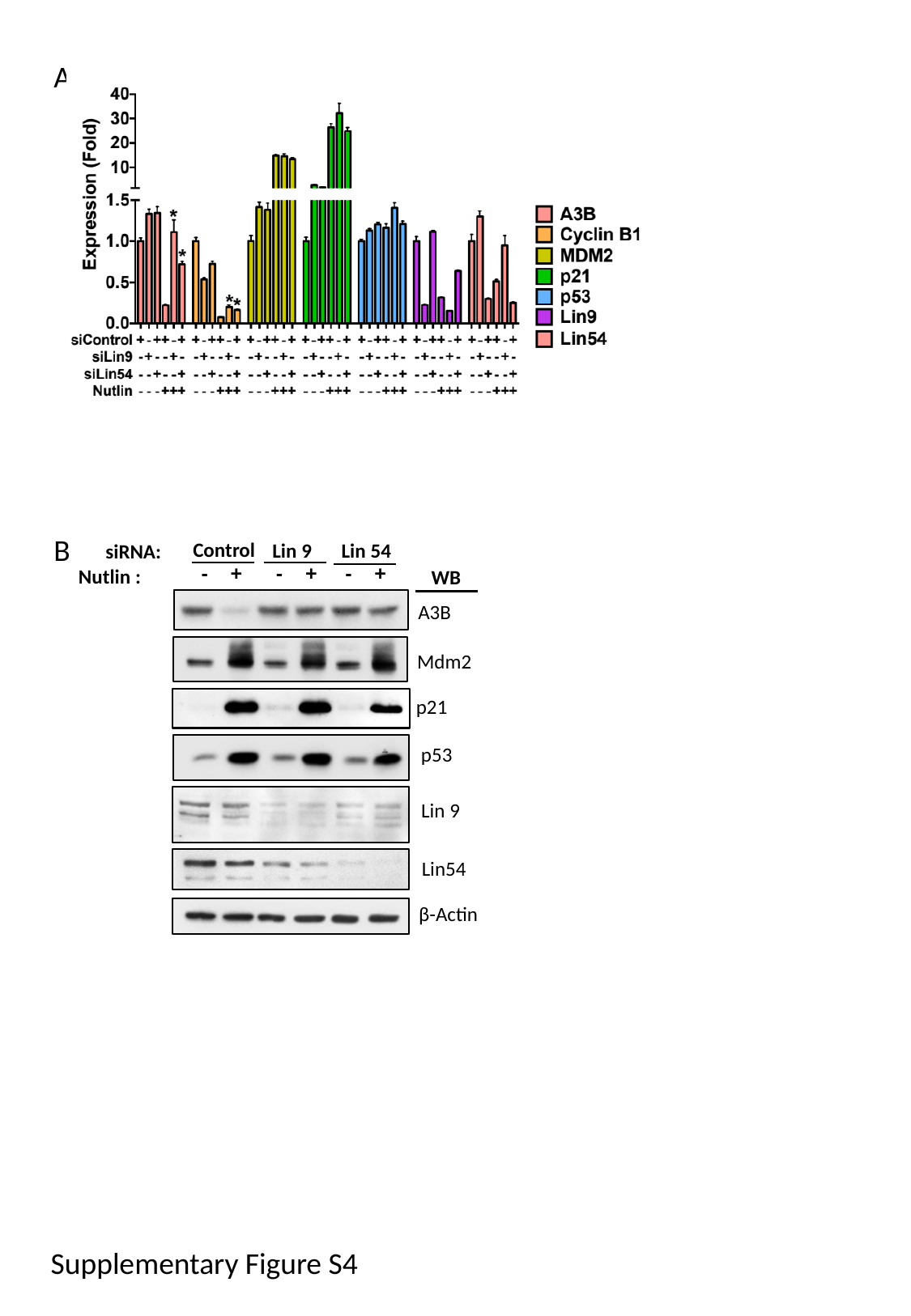

A
B
Control
Lin 9
Lin 54
siRNA:
- + - + - +
Nutlin :
WB
 A3B
Mdm2
p21
p53
Lin 9
Lin54
β-Actin
Supplementary Figure S4

## Slide 5
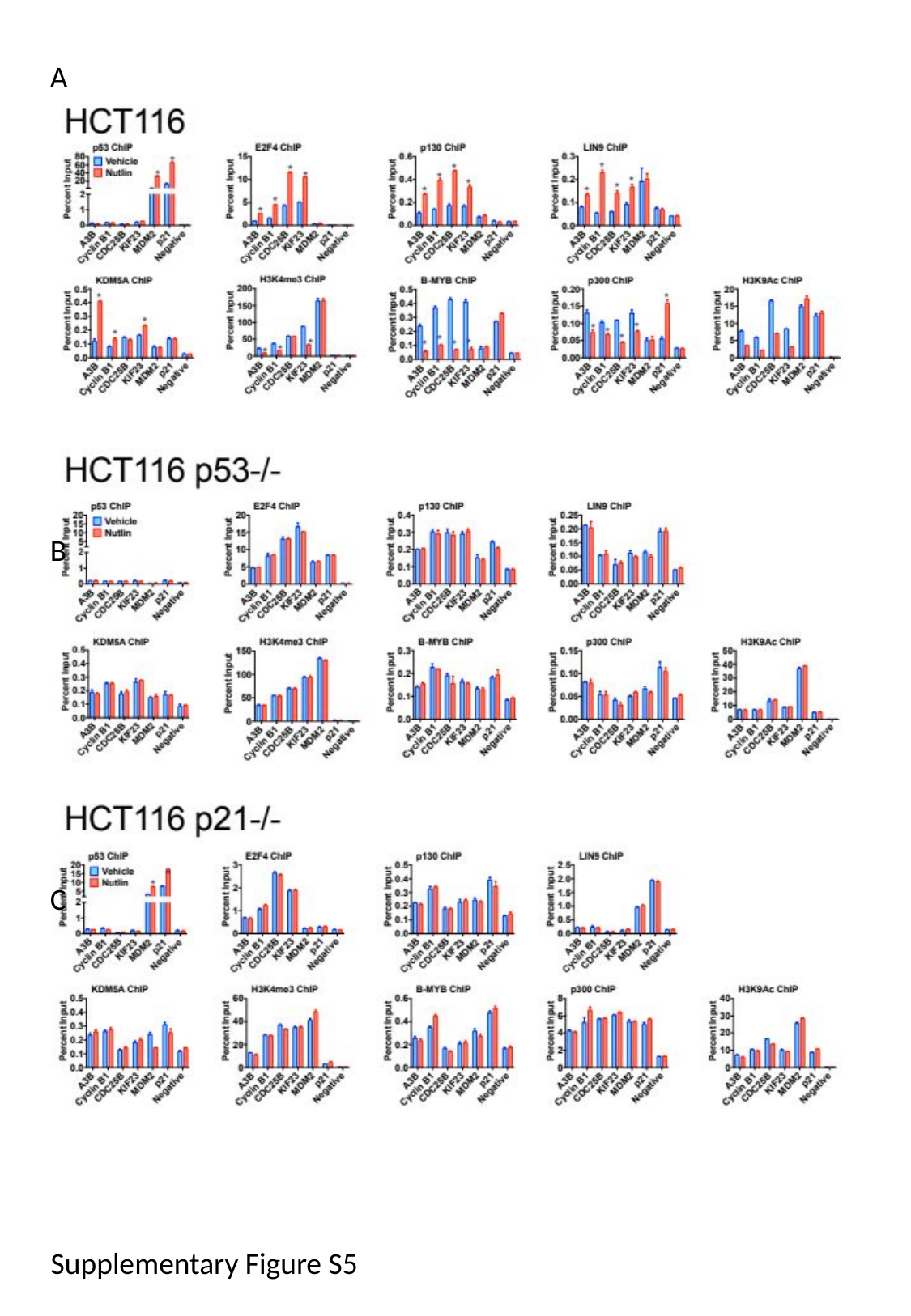

A
B
C
Supplementary Figure S5

## Slide 6
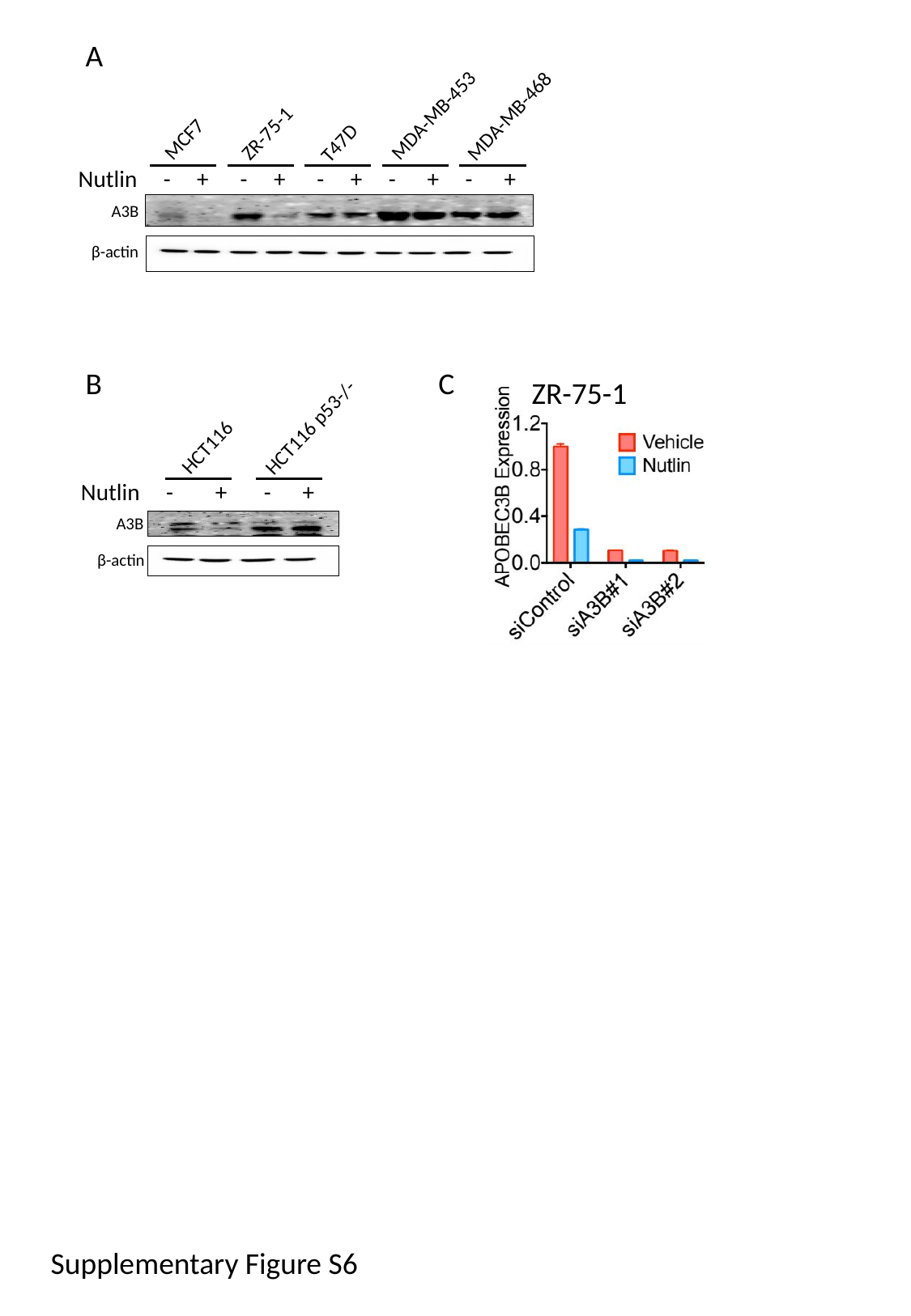

A
B C
MDA-MB-453
MDA-MB-468
ZR-75-1
MCF7
T47D
A3B
β-actin
Nutlin - + - + - + - + - +
HCT116 p53-/-
HCT116
Nutlin - + - +
A3B
β-actin
ZR-75-1
Supplementary Figure S6

## Slide 7
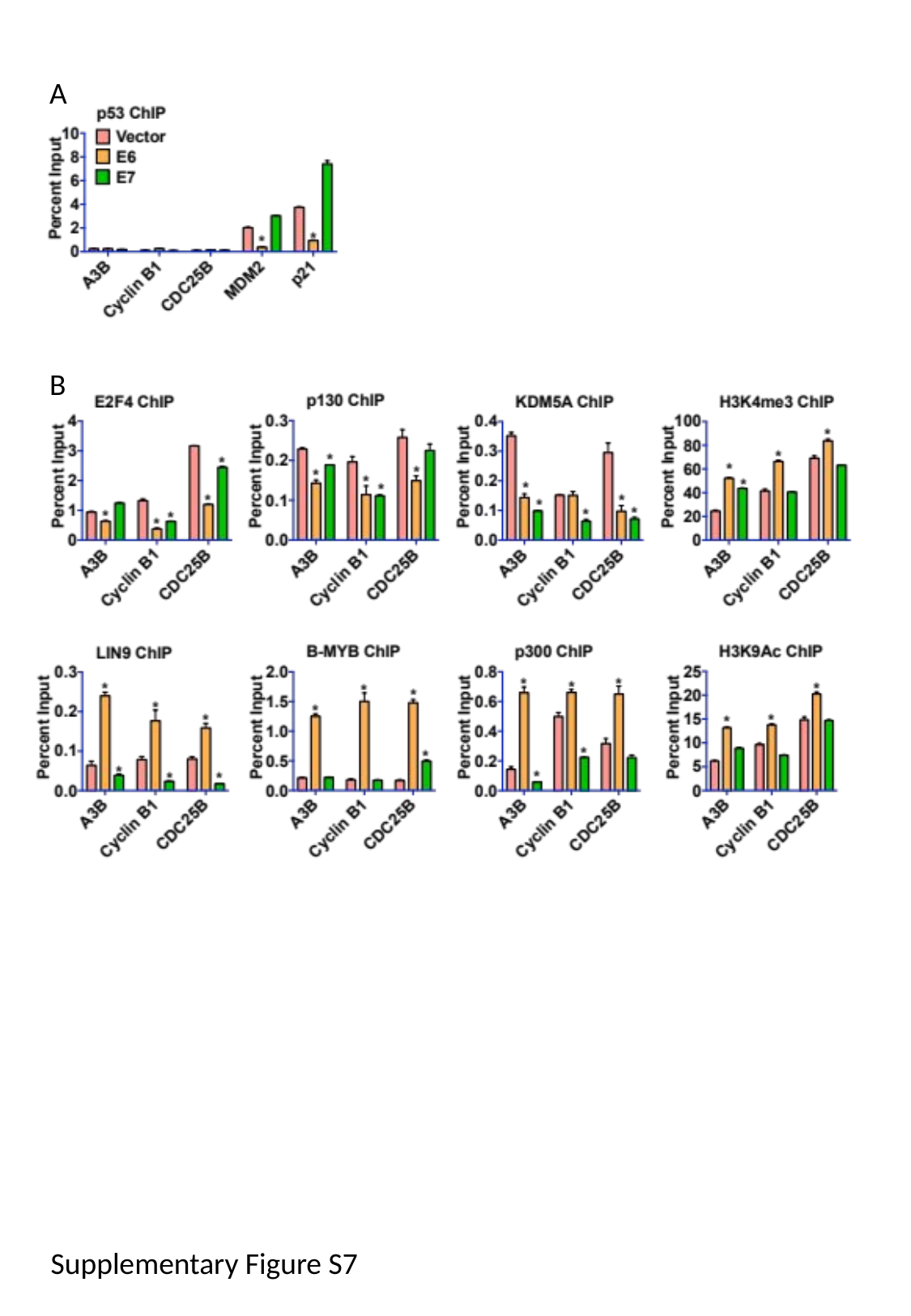

A
B
Supplementary Figure S7
